# Cost-effectiveness of an Emergency Department–Based Intensive Care Unit

**DOI:** 10.1001/jamanetworkopen.2022.33649

**Published:** 2022-09-28

**Authors:** Benjamin S. Bassin, Nathan L. Haas, Nana Sefa, Richard Medlin, Timothy A. Peterson, Kyle Gunnerson, Steve Maxwell, James A. Cranford, Stephanie Laurinec, Christine Olis, Renee Havey, Robert Loof, Patrick Dunn, Debra Burrum, Jennifer Gegenheimer-Holmes, Robert W. Neumar

**Affiliations:** 1 Division of Critical Care, Department of Emergency Medicine, University of Michigan, Ann Arbor; 2 Max Harry Weil Institute for Critical Care Research and Innovation, Ann Arbor, Michigan; 3 Department of Critical Care, Medstar Washington Hospital Center, Washington, DC; 4 Department of Emergency Medicine and Learning Health Sciences, University of Michigan, Ann Arbor; 5 Department of Emergency Medicine, University of Michigan, Ann Arbor; 6 Clinical Financial Planning & Analysis, University of Michigan, Ann Arbor

## Abstract

**Importance:**

Value in health care is quality per unit cost (V = Q/C), and an emergency department–based intensive care unit (ED-ICU) model has been associated with improved quality. To assess the value of this care delivery model, it is essential to determine the incremental direct cost of care.

**Objective:**

To determine the association of an ED-ICU with inflation-adjusted change in mean direct cost of care, net revenue, and direct margin per ED patient encounter.

**Design, Setting, and Participants:**

This retrospective economic analysis evaluated the cost of care delivery to patients in the ED before and after deployment of the Joyce and Don Massey Family Foundation Emergency Critical Care Center, an ED-ICU, on February 16, 2015, at a large academic medical center in the US with approximately 75 000 adult ED visits per year. The pre–ED-ICU cohort was defined as all documented ED visits by patients 18 years or older with a complete financial record from September 8, 2012, through June 30, 2014 (660 days); the post–ED-ICU cohort, all visits from July 1, 2015, through April 21, 2017 (660 days). Fiscal year 2015 was excluded from analysis to phase in the new care model. Statistical analysis was performed March 1 through December 30, 2021.

**Exposures:**

Implementation of an ED-ICU.

**Main Outcomes and Measures:**

Inflation-adjusted direct cost of care, net revenue, and direct margin per patient encounter in the ED.

**Results:**

A total of 234 884 ED visits during the study period were analyzed, with 115 052 patients (54.7% women) in the pre–ED-ICU cohort and 119 832 patients (54.5% women) in the post–ED-ICU cohort. The post–ED-ICU cohort was older (mean [SD] age, 49.1 [19.9] vs 47.8 [19.6] years; *P* < .001), required more intensive respiratory support (2.2% vs 1.1%; *P* < .001) and more vasopressor use (0.5% vs 0.2%; *P* < .001), and had a higher overall case mix index (mean [SD], 1.7 [2.0] vs 1.5 [1.7]; *P* < .001). Implementation of the ED-ICU was associated with similar inflation-adjusted total direct cost per ED encounter (pre–ED-ICU, mean [SD], $4875 [$15 175]; post–ED-ICU, $4877 [$17 400]; *P* = .98). Inflation-adjusted net revenue per encounter increased by 7.0% (95% CI, 3.4%-10.6%; *P* < .001), and inflation-adjusted direct margin per encounter increased by 46.6% (95% CI, 32.1%-61.2%; *P* < .001).

**Conclusions and Relevance:**

Implementation of an ED-ICU was associated with no significant change in inflation-adjusted total direct cost per ED encounter. Holding delivery costs constant while improving quality demonstrates improved value via the ED-ICU model of care.

## Key Points

**Question:**

What is the association of an emergency department–based intensive care unit (ED-ICU) with cost of care delivery?

**Findings:**

In this economic analysis performed at an academic medical center in the US, implementation of an ED-ICU was not associated with a change in inflation-adjusted cost per ED patient encounter.

**Meaning:**

These findings suggest that implementation of an ED-ICU model can provide higher value care by improving quality (reduced ICU use and 30-day mortality) without increasing cost.

## Teaser

This economic evaluation assesses the association of an emergency department–based intensive care unit with direct cost of care delivery among patients presenting to an emergency department.

## Introduction

During the last 2 decades, increasing acuity and volume of emergency department (ED) visits have resulted in greater demand for critical care services in the ED and intensive care units (ICUs).^1,2^ This change has coincided with a shortage of intensivists and resulted in increased boarding of patients requiring critical care in the ED.^1,2,3,4^ Boarding of critically ill patients in the ED is associated with worse patient outcomes.^5,6,7,8^ This association, together with increased need for critical care services, has led to the exploration of various ED critical care delivery models.^9^ These models include ED clinicians performing critical care for patients awaiting ICU admission, critical care consult services within EDs, and the ED-ICU model (ED-ICU).^10,11,12^ In February 2015, University of Michigan Health opened the Joyce and Don Massey Family Foundation Emergency Critical Care Center, an ED-ICU.

Gunnerson et al^13^ previously reported the association of ED-ICU implementation with improved quality as evidenced by improved 30-day survival and reduced inpatient ICU admissions for all patients in the ED. An editorial by Kurz and Hess^14^ suggested that without data to determine the value of this model, the feasibility and sustainability of its widespread adoption are largely uncertain. Value in health care is defined as quality per unit cost (V = Q/C).^15^ Because the ED-ICU improvement in quality has been described previously, it is essential to determine cost to assess the model’s value. The primary objective of this study was to assess the association of an ED-ICU with changes in the direct cost of care delivery (direct costs incurred by the institution to deliver patient care) to the ED and our hospital system as a whole. Additional outcomes included net revenue, net direct margin (net revenue minus cost), and professional billing for ED encounters. We hypothesized that the coordination of early high-intensity care for patients with high-acuity visits in the ED-ICU would improve care, resulting in downstream delivery cost savings to the hospital system.

## Methods

This is a retrospective economic analysis of the cost of care delivery to patients before and after implementation of the Emergency Critical Care Center, an ED-ICU at an academic medical center in the US. The institutional review board at the University of Michigan reviewed and approved this study, which included a waiver of Health Insurance Portability and Accountability Act authorization. This study retrospectively analyzed data previously collected during the course of routine clinical care and is reported in compliance with the Consolidated Health Economic Evaluation Reporting Standards (CHEERS) guideline.

The context and background of clinical operations in the University of Michigan adult ED before and after ED-ICU implementation were discussed previously by Gunnerson et al.^13^ Before ED-ICU implementation, all patients requiring ongoing critical care continued to be treated by the ED team in consultation with inpatient ICU teams. This care was continued until an inpatient ICU bed became available or the patient no longer required critical care and was admitted to a non-ICU level of care. After ED-ICU implementation, patients requiring ongoing critical care could be transferred to the ED-ICU team and cared for in the 9-bed ED-ICU, regardless of inpatient ICU bed availability.

Our hospital system, like most in the US, uses a volume-based costing model rather than prospectively capturing individualized direct patient-level costs of care. We calculated the direct cost of care for every ED patient encounter in each cohort by using the ratio of cost to charges (RCCs) and total charges for that encounter.^16,17^ Both the pre–ED-ICU and post–ED-ICU direct facility costs were estimated using the RCCs and subsequently adjusted for inflation. By using the same RCC method to derive cost data while adjusting for variables that are known to affect cost, changes in direct facility costs seen between the pre–ED-ICU and post–ED-ICU cohorts are potentially associated with implementation of the unit itself. Facility costs were analyzed given that the focus was on costs at the ED and hospital level.

**Figure 1.**
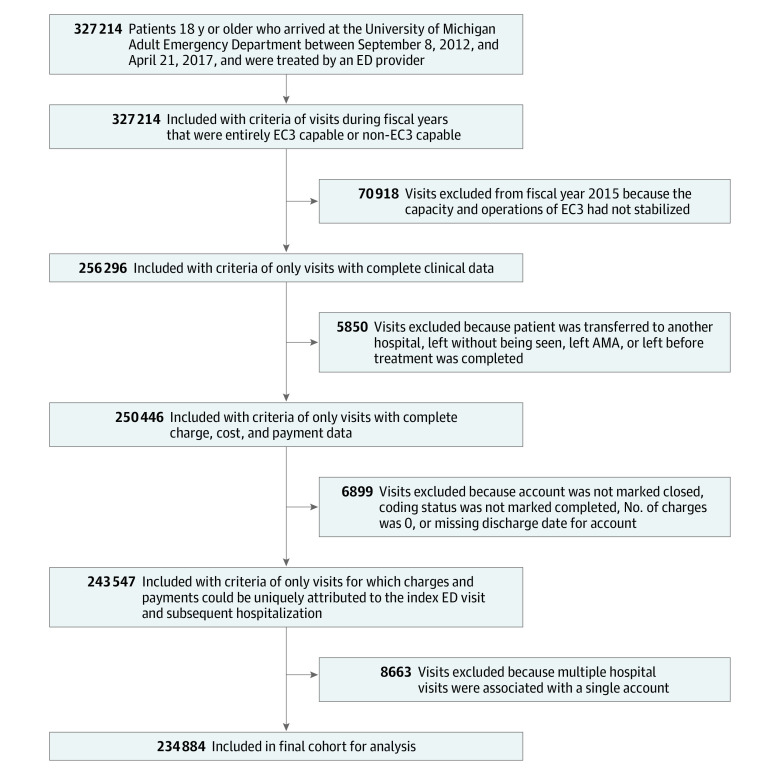
Flowchart of Study Cohort AMA indicates against medical advice; EC3, Emergency Critical Care Center; and ED, emergency department.

To test for differences in cost of care delivery before and after ED-ICU implementation, we analyzed data from the electronic health records of all ED visits from September 8, 2012, through April 21, 2017. We excluded July 1, 2014, through June 30, 2015, from analysis to allow for a washout of data over a fiscal year as we transitioned between the 2 models of care being analyzed (the ED-ICU underwent a phased opening between February and May 2015). The pre–ED-ICU cohort included all visits to the ED from September 8, 2012, through June 30, 2014 (660 days), and the post–ED-ICU cohort included all visits to the ED from July 1, 2015, through April 21, 2017 (660 days). The study data included all ED visit–associated accounts in which the patient was 18 years or older at the time of service, was treated by an ED clinician, and had a complete and interpretable financial record for the encounter. Accounts with noninterpretable financial data included accounts that (1) were still open and had a nonzero balance, (2) had no documented charges, (3) combined multiple hospitalizations (eg, index hospitalization was a scheduled procedure followed by an ED visit after discharge), (4) did not include a complete acute care hospitalization record (eg, patients who were transferred to another acute care facility during the course of their hospitalization), or (5) contained conflicts between clinical and financial records that were implausible (eg, billing from incorrect fiscal year). Rates of missing data were low, as outlined in Figure 1.

### Exposure and Outcomes

Implementation of the ED-ICU, designed to provide rapid initiation of ICU-level care in the ED and to facilitate seamless transition to inpatient ICUs, constituted the study exposure. The primary outcome of this study was the change in inflation-adjusted total direct cost per ED encounter before and after ED-ICU implementation. A subanalysis was performed to determine the direct cost of care attributed to the ED portion compared with the inpatient hospitalization portion of the encounter. Secondary outcomes included change in inflation-adjusted net revenue (payment) per patient encounter before and after ED-ICU implementation and change in inflation-adjusted direct margin (net revenue minus cost) per patient encounter among all patients in the ED before and after ED-ICU implementation.

We used the Emergency Severity Index (ESI)^18^ and the Charlson Comorbidity Index (CCI)^19^ to adjust the results for disease severity variation between pre–ED-ICU and post–ED-ICU cohorts (eTable 1 in the Supplement). The ESI represents 5 different levels of anticipated intensity of resource use based on severity of illness at ED presentation. Levels range from ESI 1 (most resource intensive) to ESI 5 (least resource intensive).

A subanalysis was performed of only patients with critical illness, defined as those admitted to the inpatient ICU in the pre–ED-ICU cohort and those transferred to the ED-ICU or admitted to the inpatient ICU in the post–ED-ICU cohort (eTable 2 in the Supplement), to determine the association of costs among the population most likely to benefit from the intervention. A further subanalysis was performed with a low-acuity subpopulation of patient encounters in the ED defined as ESI 4 and 5. Most patients with ESI 4 and 5 encounters will not require critical care and provide a comparator population that does not use the ED-ICU. This subanalysis was performed to evaluate whether unrelated temporal trends contributed to changes in cost, revenue, and margin rather than the results being associated with the intervention itself.

In addition, the impact of ED-ICU deployment on professional billing fees associated with the measured outcomes for ED encounters was quantified through the analysis of relative value units (RVUs) across the pre–ED-ICU and post–ED-ICU cohorts (eTable 3 in the Supplement). Relative value units are used to quantify the billing of physician services that can be compared across medical disciplines.^20^ We compared RVU per ED encounter across pre–ED-ICU and post–ED-ICU cohorts to determine how overall ED professional billing changed over time and whether any fundamental shifts in billing or coding practices might account for the outcomes observed. In addition, RVU per faculty hour was analyzed to assess the association with increased physician staffing required to run the ED-ICU.

### Statistical Analysis

Data from a total of 234 884 ED visits were identified and analyzed. Bivariate linear regression analyses^21^ were used to test hypotheses about pre–ED-ICU and post–ED-ICU cohort differences in (1) mean direct cost of the encounter to the hospital and (2) mean direct cost of the encounter to the ED. All cost variables were inflation-adjusted to 2018 dollars. Inflation normalization removes the impact of the inflationary component on pricing. Unlike other sectors, there is no established index for cost of care delivery inflation in the health care industry. A 4% total expense inflation assumption is a common value used for business budgeting in our organization. Personnel (nursing and physician) costs reflect most of the cost increase. Wage and benefit expenses increased 3% per year on average and larger increases were seen in pharmaceutical and other supply expenses. Therefore, 4% was used to normalize or remove the impact of inflationary price changes on the direct costs of care delivery for fiscal years 2013 to 2017.

Cluster-robust SEs were estimated to account for multiple visits clustered within patients. An α < .05 was used for all analyses, and all hypotheses were 2 sided. Analyses were conducted with Stata, version 15 (StataCorp LLC), from March 1 to December 30, 2021.

## Results

A total of 234 884 ED visits with 60 848 ED hospital admissions (representing 38 477 unique patients) during the study period were analyzed, with 115 052 patients (54.7% women and 45.1% men) in the pre–ED-ICU cohort and 119 832 (54.5% women and 45.4% men) in the post–ED-ICU cohort (Table 1). Race and ethnicity data were not collected or available for analysis. The mean (SD) age of patients was 47.8 (19.6) years in the pre–ED-ICU cohort and 49.1 (19.9) years in the post–ED-ICU cohort (*P* < .001). The mean (SD) ED length of stay increased from 6.9 (5.1) hours in the pre–ED-ICU cohort to 7.8 (5.6) hours in the post–ED-ICU cohort (*P* < .001) (the ED length of stay is inclusive of ED-ICU length of stay). The admission rate was 25.4% in the pre–ED-ICU cohort and 26.4% in the post–ED-ICU cohort (*P* < .001). The post–ED-ICU cohort had a higher proportion of patients receiving intensive respiratory support (ie, mechanical ventilation, noninvasive ventilation, heated high-flow nasal cannula) (2.2% vs 1.1%; *P* < .001) and vasopressor infusion (0.5% vs 0.2%; *P* < .001) and higher overall case mix index (mean [SD], 1.7 [2.0] vs 1.5 [1.7]; *P* < .001). Despite the increased intensity of resources, the inpatient ICU admission rate was lower (2.5% vs 2.8%; *P* < .001) in the post–ED-ICU cohort.

**Table 1.** Patient Characteristics and Use of Resources in 234 884 ED Visits

Characteristic	Patient cohort		*P* value
	Pre–ED-ICU	Post–ED-ICU	
Total ED visits included in analysis, No. (%)	115 052 (49.0)	119 832 (51.0)	NA
ED visits, mean No. per y	63 574.5	66 215.8	NA
ED visits treated in EC3, No. (%)	NA	4158 (3.5)	NA
Age, mean (SD), y	47.8 (19.6)	49.1 (19.9)	<.001
Legal sex, No. (%)			
Women	62 998 (54.7)	65 331 (54.5)	.25
Men	51 955 (45.1)	54 395 (45.4)	.25
Unknown	99 (0.1)	106 (0.1)	.84
Triaged to ED resuscitation bay, No. (%)	5991 (5.2)	8343 (7.0)	<.001
ED LOS, mean (SD), h	6.9 (5.1)	7.8 (5.6)	<.001
EC3 LOS, mean (SD), h^a^	NA	12.9 (7.6)	
Admitted to hospital, No. (%)	29 195 (25.4)	31 653 (26.4)	<.001
ED-ICU visit requirements, No. (%)^a^			
Mechanical ventilation	NA	1203 (28.9)	NA
Vasopressors, No. (%)	NA	456 (11.0)	NA
Respiratory support, No. (%)^b^	1234 (1.1)	2683 (2.2)	<.001
Vasopressors, No. (%)	202 (0.2)	543 (0.5)	<.001
Case mix index, mean (SD)^c^	1.5 (1.7)	1.7 (2.0)	<.001
Admitted to ICU, No. (%)	3252 (2.8)	2951 (2.5)	<.001
Billed ICU LOS, mean (SD), d	4.5 (7.0)	4.8 (7.9)	.07
ICU LOS, mean (SD), d	4.8 (7.5)	5.2 (7.7)	.01
Readmission within 72 h of discharge, No. (%)	592 (0.5)	706 (0.6)	.03

Abbreviations: EC3, Emergency Critical Care Center; ED-ICU, emergency department–intensive care unit; LOS, length of stay; NA, not applicable.

^a^ Based on 4158 EC3 visits.

^b^ Includes heated high-flow nasal cannula, noninvasive ventilation (bilevel positive airway pressure, continuous positive airway pressure), and mechanical ventilation.

^c^ Calculated as the mean of the diagnosis-related group relative weights for inpatient discharge cases.

Total direct costs per ED encounter were similar in the pre–ED-ICU (mean [SD], $4875 [$15 175]; median, $1110 [range, $0.01-$1 222 961]; total, $560 573 021) and post–ED-ICU (mean [SD], $4877 [$17 400]; median, $1138 [range, $0.59-$3 279 953]; total, $584 443 676) cohorts (change, 0.04% [95% CI, −2.7% to 2.8%]; *P* = .98).There was a statistically significant increase in direct ED cost per ED encounter (mean [SD], $660 [$669] vs $717 [$959]; change, 8.6% [95% CI, 7.6%-9.7%]; *P* < .001) and a nonstatistically significant decrease in direct hospital cost per ED encounter (mean [SD], $4216 [$14 997] vs $4161 [$17 187]; change, −1.3% [95% CI, −4.4% to 1.8%]; *P* = .44). Net revenue per encounter increased from the pre–ED-ICU to the post–ED-ICU cohorts (mean [SD], $5728 [$20 151] vs $6132 [$28 839]; change, 7.0% [95% CI, 3.5%-10.6%]; *P* < .001). Similarly, direct margin per encounter increased (mean [SD], $856 [$10 739] vs $1255 [$14 987]; change, 46.6% [95% CI, 32.1%-61.2%]; *P* < .001) (Table 2).

**Table 2.** Inflation-Adjusted Pre–ED-ICU and Post–ED-ICU Financial Metrics for All Patients Presenting to ED^a^

Variable	Patient cohort^**b**^		*P* value	Change (95% CI), %
	Pre–ED-ICU	Post–ED-ICU		
Total cases, No. (%)	115 052 (49.0)	119 832 (51.0)	NA	NA
Total direct costs per ED encounter, $	4875 (15 175)	4877 (17 400)	.98	0.04 (–2.7 to 2.8)
Direct ED cost per ED encounter	660 (669)	717 (959)	<.001	8.6 (7.6 to 9.7)
Direct hospital cost per ED encounter	4216 (14 997)	4161 (17 187)	.44	–1.3 (–4.4 to 1.8)
Total charges, $	15 574 (46 094)	17 297 (54 972)	<.001	11.1 (8.3 to 13.8)
ED charges, $	2503 (2521)	2994 (4010)	<.001	19.6 (18.4 to 20.7)
ED charges, % of total charges	16.1	17.3	<.001	7.4 (5.5 to 9.4)
Total net revenue, $	5728 (20 151)	6132 (28 839)	<.001	7.0 (3.5 to 10.6)
Total direct margin, $	856 (10 739)	1255 (14 987)	<.001	46.6 (32.1 to 61.2)

Abbreviations: ED-ICU, emergency department–intensive care unit; NA, not applicable.

^a^ Pre–ED-ICU discharge fiscal year (FY) includes FY2013 (FY2013 cases: September 2012 to June 30, 2013; September 2012 represents a partial month) and FY2014. Post–ED-ICU discharge includes FY2016 and FY2017 (FY2017 cases: July 1, 2016, to April 2017; April 2017 represents a partial month).

^b^ Unless indicated otherwise, data are expressed as mean (SD).

Total direct cost per ED encounter remained unchanged when adjusted for CCI (change, −0.07% [95% CI, −3.6% to 2.3%]; *P* = .63) and decreased when adjusted for ESI (change, −4.5% [95% CI, −7.3% to 1.8%]; *P* = .001). Total net revenue per case increased when adjusted for both CCI (change, 6.4% [95% CI, 2.5%-10.2%]; *P* = .001) and ESI (change, 2.0% [95% CI, −1.6% to 5.7%]; *P* = .26). Total direct margin per case also increased when adjusted for both CCI (change, 46.3% [95% CI, 30.5%-62.2%]; *P* < .001) and ESI (change, 38.9% [95% CI, 23.9%-54.0%]; *P* < .001) (eTable 1 in the Supplement).

The subanalysis of critically ill patients (9908 [4.2% of total ED encounters]) demonstrated cost savings for this subpopulation, with total direct cost per ED encounter decreasing by 22.1% (95% CI, −26.8% to −17.5%; *P* < .001). Total net revenue decreased by 19.5% (95% CI, −25.4% to −13.5%; *P* < .001). Because both total direct cost and net revenue decreased, total direct margin had no statistically significant change, but remained positive (mean [SD], $7841 [$47 624] vs $7290 [$36 001]; *P* = .52) (eTable 2 in Supplement).

In a subanalysis of patients with low-acuity visits (ESI 4 and 5) (25 863 [11.0% of total ED encounters]), total direct cost per ED encounter was unchanged between pre–ED-ICU and post–ED-ICU cohorts (mean [SD], $533 [$2085] vs $525 [$1679]; change, −1.5% [95% CI, −10.2% to 7.1%]; *P* = .74). However, increases in both total net revenue (mean [SD], $708 [$2116] vs $782 [$2585]; change, 10.4% [95% CI, 1.9%-19.0%]; *P* = .01) and total direct margin (mean [SD], $175 [$1377] vs $257 [$1679]; change, 46.9% [95% CI, 19.1%-74.6%]; *P* < .001) were found in this population (eTable 2 in the Supplement).

Analysis of overall RVUs per encounter increased significantly between pre–ED-ICU and post–ED-ICU cohorts when ED-ICU RVUs were included (2.94 vs 3.15; 7.1% increase; *P* < .001). The portion attributed to ED-ICU billing was 5.47 RVUs per encounter for ED-ICU encounters only. However, the total did not change significantly across the entire ED population in the pre–ED-ICU– vs post–ED-ICU cohorts when ED-ICU encounters were excluded (2.94 vs 2.96 RVU per encounter). Relative value units per attending hour decreased by 9.4% between the pre–ED-ICU and post–ED-ICU cohorts (6.55 vs 5.94; *P* < .001) (eTable 3 in Supplement).

## Discussion

Implementation of an ED-ICU has previously been associated with improved patient outcomes (15.4% reduction in risk-adjusted 30-day mortality) and use of resources (12.9% reduction in ICU admission).^13^ In this study, we demonstrate the inflation-adjusted total direct cost per ED encounter remained unchanged despite an 8.6% increase in direct ED cost per ED encounter. The ability to hold overall cost per ED encounter constant while caring for a patient cohort with higher-acuity, more resource-intensive needs and improving patient outcomes could be attributable to the implementation of the ED-ICU.

The increased direct cost to the ED is not unexpected, because the infrastructure and staff required to operate an ED-ICU and extended critical care provision is resource intensive. The largest portion of these increased costs is secondary to labor with dedicated staffing of nurses, a respiratory therapist, a combination of nontrainee (physician assistants) and trainee (residents and fellows) staffing, and an attending physician 24 hours per day. Physician assistants represent a mean of 28.5% of nonattending ED-ICU staffing, whereas residents and fellows represent the other 71.5%. The ratio of trainee to nontrainee staffing and associated staffing expense is likely to vary based on multiple local and institutional factors. In addition, increased diagnostic testing and therapeutic interventions within the ED-ICU likely increased overall ED costs to deliver care. However, the ED costs per encounter only represented 14.7% of the overall cost per encounter and increased by only a mean of $57 per case ($660 vs $717; *P* < .001), whereas the inpatient cost per encounter decreased by a mean of $55 per case ($4216 vs $4161; *P* = .44). Improved quality of ED care potentially provided downstream care delivery cost savings and allowed the intervention to maintain overall cost neutrality.

Implementation of an ED-ICU was associated with overall cost reduction per encounter for the subpopulation of patients receiving ICU-level care (22.1%; *P* < .001). In contrast, there was no overall cost reduction for low-acuity ED encounters defined as ESI 4 and 5 (1.5%; *P* = .74). We hypothesize that these observed findings of reduced costs for critically ill patients are associated with early, coordinated critical care delivery in an ED-ICU when the need is identified, rather than when an ICU bed is available. Prior studies^22^ have demonstrated increased morbidity and mortality across disease states for boarding critically ill patients in the ED. By initiating critical care interventions early in the ED-ICU, we hypothesize that progression of disease severity and complications due to delayed ICU-level care during ED boarding were avoided, resulting in improved downstream patient outcomes with associated overall cost reductions.

Analysis of overall RVUs per encounter increased significantly when ED-ICU RVUs were included (2.94 vs 3.15; 7.1% increase; *P* < .001). However, this finding is expected because the provision of longitudinal critical care results in additional billing for 99291 and 99292 emergency medicine critical care codes. Patients in the ED-ICU generated a mean of 5.47 RVUs per encounter for ED-ICU encounters. However, total RVUs per encounter did not change significantly across the entire population in the ED in the pre–ED-ICU vs post–ED-ICU cohorts when ED-ICU encounters were excluded (2.94 vs 2.96 RVUs per encounter). This finding suggests that no fundamental shift in our ED billing and coding or documentation practices affected our revenue over time between the 2 cohorts. In addition, we found a significant decrease (−9.4%; *P* < .001) in RVUs per attending hour between the pre–ED-ICU and post–ED-ICU cohorts, which illustrates that attending physician hourly billing during ED-ICU staffing was lower than billing during main ED staffing.

**Figure 2.**
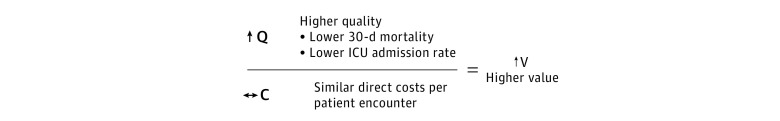
Completing the Value Equation for the Emergency Department (ED)–Based Intensive Care Unit (ICU)

This analysis reports the direct ED and hospital system costs associated with a novel care delivery mechanism (the ED-ICU), allowing completion of the value equation when paired with prior quality data (Figure 2).^13^ Previous work^23,24,25,26,27,28,29,30,31,32^ has investigated the impact of other novel care delivery mechanisms on component(s) of value in emergency medicine, including telehealth, clinician in triage, split flow, and discharge lounges. The use of telehealth in EDs has been associated with estimated lower total annual ED costs and improved quality via reduced ED length of stay and waiting time.^29,32^ Conversely, clinician-in-triage models have been associated with improved quality via reduced ED crowding, ED length of stay, and rate of leaving without being seen but also with increased costs to EDs and lack of cost-effectiveness.^23,24,25,26,27^ Split-flow models and discharge lounges have been linked to improved quality via improved efficiency, reductions in ED length of stay, and reductions in ED crowding,^27,28,29,30,31^ although to our knowledge, assessments of costs (and thereby value) are lacking. Our findings add to this body of literature by providing costs associated with implementing an ED-ICU to complete the assessment of value. With growing interest in transitioning to high-value health care,^33,34,35,36^ individuals and institutions contemplating implementation of a novel care delivery mechanism should consider available quality and cost data to best guide assessments of potential value added.

Future research should investigate the value the ED-ICU model creates for health care payers, including insurance companies, society, and patients. Conceptually, the reduced need for ICU and inpatient care is likely financially beneficial to payers. However, it is not known whether or how the increased ICU capacity generated by an ED-ICU impacts overall health care payments for an insured population. Although overall net revenue and total direct margin for ED patient care increased in the post–ED-ICU period, it is unclear whether these increases can be attributed directly to ED-ICU implementation. The direct charges for patients ultimately admitted to the ICU decreased (eTable 2 in the Supplement) in the post–ED-ICU period, whereas margin for this subpopulation was unchanged, suggesting that the source of increased revenue and margin came from the care of ED patients not admitted to the ICU. Although the revenue and margin improvements could be attributed to care of patients in the ED-ICU who were not admitted to an inpatient ICU, they could also be attributed to treatment of non–critically ill patients in the ED in the post–ED-ICU period. In fact, subanalysis of ESI 4 and 5 encounters indicates increased revenue and margin for this patient population in the post–ED-ICU period.

### Limitations

This study has some limitations. As a retrospective study with a before-and-after analysis at a single center, external generalizability is unknown. Before-and-after analyses are limited to assessment of association rather than direct causation, and additional unaccounted confounders (including temporal trends) may impact the observed results. The associated costs, revenue, margin, and sustainability are likely to differ at institutions with different staffing expenses (including those with different ratios of trainees [resident physicians or fellows] vs nontrainees), payer mixes, and inpatient capacity constraints. It is also possible that overuse or unnecessary use of the ED-ICU could lead to increased costs in other settings. Net revenue and total direct margin increased during the study period despite a higher degree of acuity in the post–ED-ICU cohort, as manifested by older age, higher case mix index, and higher rate of requirements for respiratory support or vasopressors, although this may have been confounded by margin attributed to a portion of patients that were not critically ill (eTable 2 in the Supplement).

Our analysis did not specifically account for construction or planning expenses, although infrastructure depreciation is a part of the expense included in our analysis. This start-up process and associated expenses are likely to vary by institution and local factors (eg, only altering staffing models vs complete design and construction of a new facility), and thus we focused our financial analysis instead on operating expenses once implemented. Those considering implementing an ED-ICU at their institution should consider local circumstances with the expense and revenue data presented in this study.

Our evaluation of cost and its components analyzes the total direct facility costs to the hospital system for pre–ED-ICU and post–ED-ICU cohorts. Direct facility costs constitute a myriad of elements, including labor, pharmaceuticals, and supplies to facilitate patient care. Direct facility costs for the hospital system are driven by the institution’s contracts to support required staffing and other resources. The relatively flat total direct inflation-adjusted ED and hospital costs may reflect effective cost management practices across the health care system and not just those efforts to efficiently manage the ED-ICU. The health care system’s net direct margin is impacted by many elements, including contracting, case management, intensity of service, and payer mix. This financial impact study profiled the overall direct costs and net revenues and did not distinguish between components influencing margin performance. It is possible payer mix changes across measurement periods or other revenue drivers not quantified in this study contributed to the calculated margin performance.

The 4% rate of inflation used in this analysis may be an overestimate or an underestimate of actual health care delivery expense and revenue inflation in the study periods. Although this assumption is based on components of the actual experience of our health system and grounded in the annual budgeting practice of our organization, overadjusting or underadjusting for inflation could have an impact on the net margin calculation for an ED-ICU.

Also, this work is limited by data that were previously collected, although rates of missing data were low (<0.1%). The cost analysis used RCCs, which is an estimation of the cost of care delivery and not the same as more precise activity-based costing systems. This is because our hospital currently does not use activity-based costing for cost analysis, and thus data for such a system were not available. However, because we were primarily performing an analysis at a hospital system level, a macro level that encompasses the global cost of care delivery, RCCs would have a higher level of accuracy.

This evaluation examines the costs and revenues associated with a portion of the care a patient would experience in an ED and hospital for a given episode of illness. The evaluation of an ongoing investment in this model of care should consider variables impacting the total episode cost and net revenue of the patient care encounters across the spectrum of health care services provided.

## Conclusions

Previous work demonstrated that an ED-ICU was associated with improved care quality and patient outcomes via reductions in use of the ICU and 30-day mortality. Our economic analysis completes this value assessment by demonstrating that the ED-ICU model can work in tandem with the ED and hospital to provide care in a cost-effective manner. Improving quality while holding overall delivery costs constant can potentially increase the value of health care delivery.

## Notes

Supplement. **eTable 1.** Pre– and Post–ED-ICU Financial Metrics for All Patients Presenting to ED Adjusted for Inflation, Charlson Comorbidity Index (CCI), and Emergency Severity Index (ESI) **eTable 2.** Pre– and Post–ED-ICU Financial Metrics Comparison of ESI 4 and 5 vs Critically Ill Patients (Admitted to ICU or EC3) **eTable 3.** Pre– and Post–ED-ICU Cohort Comparison RVUs per Visit and RVUs per Attending Hours
