# Supplementary material for: Cost-effectiveness of an Emergency Department–Based Intensive Care Unit
Source: JAMA Netw Open. 2022 Sep 28;5(9):e2233649. doi: 10.1001/jamanetworkopen.2022.33649 (PMC9520346; doi:10.1001/jamanetworkopen.2022.33649)
Supplement: Supplement. — eTable 1. Pre– and Post–ED-ICU Financial Metrics for All Patients Presenting to ED Adjusted for Inflation, Charlson Comorbidity Index (CCI), and Emergency Severity Index (ESI) eTable 2. Pre– and Post–ED-ICU Financial Metrics Comparison of ESI 4 and 5 vs Critically Ill Patients (Admitted to ICU or EC3) eTable 3. Pre– and Post–ED-ICU Cohort Comparison RVUs per Visit and RVUs per Attending Hours [file jamanetwopen-e2233649-s001.pdf]

# Supplementary Online Content

Bassin BS, Haas NL, Sefa N, et al. Cost-effectiveness of an emergency department–based intensive care unit. *JAMA Netw Open*. 2022;5(9):e2233649. doi:10.1001/jamanetworkopen.2022.33649

**eTable 1.** Pre– and Post–ED-ICU Financial Metrics for All Patients Presenting to ED Adjusted for Inflation, Charlson Comorbidity Index (CCI), and Emergency Severity Index (ESI)
**eTable 2.** Pre– and Post–ED-ICU Financial Metrics Comparison of ESI 4 and 5 vs Critically Ill Patients (Admitted to ICU or EC3)
**eTable 3.** Pre– and Post–ED-ICU Cohort Comparison RVUs per Visit and RVUs per Attending Hours

This supplementary material has been provided by the authors to give readers additional information about their work.

© 2022 Bassin BS et al. *JAMA Network Open*.

**eTable 1. Pre– and Post–ED-ICU Financial Metrics for All Patients Presenting to ED Adjusted for Inflation, Charlson Comorbidity Index (CCI), and Emergency Severity Index (ESI)[a]**

| | **Inflation Adjusted[a]** | | | | **Inflation and CCI Adjusted[b,c]** | | | | **Inflation and ESI Adjusted[b,c]** | | | |
|---|---|---|---|---|---|---|---|---|---|---|---|---|
| | **Pre-EC3 Cohort** | **Post-EC3 Cohort** | ***p*** | **% Change (95% CI)** | **Pre-EC3 Cohort** | **Post-EC3 Cohort** | ***p*** | **% Change (95% CI)** | **Pre-EC3 Cohort** | **Post-EC3 Cohort** | ***p*** | **% Change (95% CI)** |
| Total direct cost per ED encounter, mean (SD) | $4,875 ($15,175) | $4,877 ($17,400) | 0.98 | 0.04 (-2.7, 2.8) | $4,894 ($49.8) | $4,860 ($54.6) | 0.63 | (-0.07) (-3.6, 2.3) | $4,992 ($49.2) | $4,765 ($53.3) | 0.001 | (-4.5) (-7.3, -1.8) |
| Direct ED cost per ED encounter, mean (SD) | $660 ($669) | $717 ($959) | <.001 | 8.6 (7.6, 9.7) | $661 ($2.3) | $715 ($3.5) | <.001 | 8.2 (6.9, 9.4) | $668 ($2.2) | $708 ($3.3) | <.001 | 6.0 (4.6, 7.0) |
| Direct hospital cost per ED encounter, mean (SD) | $4,216 ($14,997) | $4,161 ($17,187) | 0.44 | (-1.3) (-4.4, 1.8) | $4,232 ($39.0) | $4,136 ($53.8) | 0.22 | (-2.3) (-5.4, 1.3) | $4,324 ($48.6) | $4,058 ($52.6) | <.001 | (-6.1) (-9.3, -3.0) |
| Total Charges, mean (SD) | $15,574 ($46,094) | $17,297 ($54,972) | <.001 | 11.1 (8.3, 13.8) | $15,636 ($151.8) | $17,238 ($174.6) | <.001 | 10.2 (8.1, 12.4) | $15,957 ($149.3) | $16,929 ($169.4) | <.001 | 6.1 (4.1, 8.1) |

© 2022 Bassin BS et al. *JAMA Network Open*.

| | | | | | | | | | | | | | | |
|---|---|---|---|---|---|---|---|---|---|---|---|---|---|---|
| ED Charges, mean (SD) | $2,503 ($2,521) | $2,994 ($4,010) | <.001 | 19.6 (18.4, 20.7) | | $2,508 ($8.8) | $2,989 ($14.5) | <.001 | 19.2 (17.8, 20.6) | | $2,540 ($8.3) | $2,959 ($13.9) | <.001 | 16.5 (15.2, 17.8) |
| ED Charges/Total Charges, % | 16.1% | 17.3% | <.001 | 7.4 (5.5, 9.4) | | N/A | N/A | | | | N/A | N/A | | |
| Total Net Revenue, mean (SD) | $5,728 ($20,151) | $6,132 ($28,839) | <.001 | 7.0 (3.5, 10.6) | | $5,748 ($65.8) | $6,114 ($88.4) | 0.001 | 6.4 (2.5, 10.2) | | $5,873 ($65.1) | $5,993 ($87.3) | 0.26 | 2.0 (-1.6, 5.7) |
| Total Direct Margin, mean (SD) | $856 ($10,739) | $1,255 ($14,987) | <.001 | 46.6 (32.1, 61.2) | | $857 ($35.3) | $1,254 ($46.2) | <.001 | 46.3 (30.5, 62.2) | | $884 ($35.9) | $1,228 ($46.1) | <.001 | 38.9 (23.9, 54.0) |

[a]Pre-ED-ICU Discharge Fiscal Year: FY2013, FY2014 (FY2013 cases: 9/2012 - 6/2013); September 2012 represents a partial month
Post-ED-ICU Discharge Fiscal Year: FY2016, FY2017 (FY2017 cases: 7/2016 - 4/2017); April 2017 represents a partial month
[b]Standard errors are reported for model-based estimates adjusting for CCI and ESI
[c]N/A. Model-based estimates cannot be calculated

© 2022 Bassin BS et al. *JAMA Network Open*.

**eTable 2. Pre– and Post–ED-ICU Financial Metrics Comparison of ESI 4 and 5 vs Critically Ill Patients (Admitted to ICU or EC3)**

| | **Inflation Adjusted Admitted to ICU or EC3** | | | | **Inflation Adjusted ESI 4 and 5[b]** | | | |
|---|---|---|---|---|---|---|---|---|
| | **Pre-EC3 Cohort** | **Post-EC3 Cohort** | ***p*** | **% Change (95% CI)** | **Pre-EC3 Cohort** | **Post-EC3 Cohort** | ***p*** | **% Change (95% CI)** |
| Total Cases | 3,663 (37.0%) | 6,245 (63.0%) | | | 12,944 (50.1%) | 12,919 (49.9%) | | |
| Total direct cost per ED encounter, mean (SD) | $36,610 ($52,695) | $28,497 ($42,901) | <.001 | (-22.1) (-26.8,-17.5) | $533 ($2,085) | $525 ($1,679) | 0.74 | (-1.5) (-10.2, 7.1) |
| Direct ED cost per ED encounter, mean (SD) | $1,770 ($1,209) | $3,457 ($2,401) | <.001 | 95.3 (89.9, 100.8) | $258 ($176) | $266 ($219) | 0.002 | 3.1 (1.2, 5.0) |
| Direct hospital cost per ED encounter, mean (SD) | $34,883 ($52,648) | $25,051 ($43,093) | <.001 | (-28.2) (-32.8, -23.5) | $273 ($2,016) | $257 ($1,603) | 0.48 | (-5.9) (-21.7, 10.0) |
| Total Charges, mean (SD) | $112,138 ($158,933) | $99,670 ($144,892 ) | <.001 | (-11.1) (-16.3, -5.9) | $1,862 ($6,110) | $2,066 ($5,944) | <.001 | 11.0 (2.9, 19.0) |
| ED Charges, mean (SD) | $6,715 ($4,575) | $14,436 ($10,017) | <.001 | 115.0 (108.9, 121.0) | $981 ($665) | $1,111 ($926) | <.001 | 13.2 (11.1, 15.4) |
| ED Charges/Total Charges, % | 6.0% | 14.5% | <.001 | 141.7 (107.8, 176.5) | 52.7% | 53.8% | 0.08 | 2.1 (-0.2, 4.4) |
| Total Net Revenue, mean (SD) | $44,452 ($80,092) | $35,788 ($65,420) | <.001 | (-19.5) (-25.4, -13.5) | $708 ($2,116) | $782 ($2,585) | 0.01 | 10.4 (1.9, 19.0) |
| Total Direct Margin, mean (SD) | $7,841 ($47,624) | $7,290 ($36,001) | 0.52 | (-7.0) (-26.7, 12.6) | $175 ($1,377) | $257 ($1,679) | <.001 | 46.9 (19.1, 74.6) |

© 2022 Bassin BS et al. *JAMA Network Open*.

[a]Pre- EC3 Discharge Fiscal Year: FY2013, FY2014 (FY2013 cases: 9/2012 – 6/30/2013); September 2012 represents a partial month.
Post- EC3 Discharge Fiscal Year: FY2016, FY2017 (FY2017 cases: 7/1/2016 – 4/2017); April 2017 represents a partial month.
FY2015 is the launch year for EC3 and is excluded.
[b]Standard errors are reported for model-based estimates adjusting for CCI and ESI

© 2022 Bassin BS et al. *JAMA Network Open*.

**eTable 3. Pre– and Post–ED-ICU Cohort Comparison RVUs per Visit and RVUs per Attending Hours**

| | Adult ED | | | ED-ICU | | | Adult ED + ED-ICU | | |
|---|---|---|---|---|---|---|---|---|---|
| | Visits | RVU's[1] | Hours[2] | Visits | RVU's | Hours | Visits | RVU's | Hours |
| FY13 | 70,634 | 202,416 | 31,184 | | | | 70,634 | 202,416 | 31,184 |
| FY14 | 69,743 | 209,688 | 31,746 | | | | 69,743 | 209,688 | 31,746 |
| FY16 | 74,964 | 221,303 | 30,856 | 2,561 | 13,973 | 8,784 | 74,964 | 235,276 | 39,640 |
| FY17 | 74,999 | 222,670 | 31,053 | 2,500 | 13,704 | 8,760 | 74,999 | 236,374 | 39,813 |
| | | | | | | | | | |
| FY13 and 14 | 140,377 | 412,104 | 62,930 | - | - | - | 140,377 | 412,104 | 62,930 |
| FY16 and 17 | 149,963 | 443,973 | 61,909 | 5,061 | 27,677 | 17,544 | 149,963 | 471,650 | 79,453 |
| Change | | | | | | | 6.8% | 14.4% | 26.3% |
| | | | | | | | | | |
| | | | | | | | | | |
| | Adult ED | | | ED-ICU | | | Adult ED + ED-ICU | | |
| | | RVU's/Visit | RVU's/Hour | | RVU's/Visit | RVU's/Hour | | RVU's/Visit | RVU's/Hour |
| FY13 | | 2.87 | 6.49 | | | | | 2.87 | 6.49 |
| FY14 | | 3.01 | 6.61 | | | | | 3.01 | 6.61 |

© 2022 Bassin BS et al. *JAMA Network Open*.

| | | | | | | | | | |
|---|---|---|---|---|---|---|---|---|---|
| **FY16** | | **2.95** | **7.17** | | **5.46** | **1.59** | | **3.14** | **5.94** |
| **FY17** | | **2.97** | **7.17** | | **5.48** | **1.56** | | **3.15** | **5.94** |
| | | | | | | | | | |
| **FY13 and 14*** | | **2.94** | **6.55** | | | | | **2.94** | **6.55** |
| **FY16 and 17**** | | **2.96** | **7.17** | | **5.47** | **1.58** | | **3.15** | **5.94** |
| **Change** | | | | | | | | **7.1%** | **-9.4%** |
| | | | | | | | | | |
| **[1]RVUs = Relative Value Units** | | | | | | | | | |
| **[2]Hours = Attending Faculty Hours of Coverage** | | | | | | | | | |
| ***FY13+FY14 = Pre–ED-ICU Cohort** | | | | | | | | | |
| ****FY16+FY17 = Post–ED-ICU Cohort** | | | | | | | | | |

© 2022 Bassin BS et al. *JAMA Network Open*.
